# SARS-CoV-2 Aptasensors Based on Electrochemical Impedance Spectroscopy and Low-Cost Gold Electrode Substrates

**DOI:** 10.1021/acs.analchem.1c04456

**Published:** 2022-01-19

**Authors:** Perrine Lasserre, Banushan Balansethupathy, Vincent J. Vezza, Adrian Butterworth, Alexander Macdonald, Ewen O. Blair, Liam McAteer, Stuart Hannah, Andrew C. Ward, Paul A. Hoskisson, Alistair Longmuir, Steven Setford, Eoghan C. W. Farmer, Michael E. Murphy, Harriet Flynn, Damion K. Corrigan

**Affiliations:** † Department of Biomedical Engineering, University of Strathclyde, 106 Rottenrow East, Glasgow G4 0NW, U.K.; ‡ Aptamer Group, Suite 2.78−2.91, Bio Centre, Innovation Way, Heslington, York YO10 5NY, U.K.; § Department of Civil and Environmental Engineering, University of Strathclyde, 75 Montrose Street, Glasgow G1 1XJ, U.K.; ∥ Strathclyde Institute of Pharmacy and Biomedical Sciences (SIPBS), University of Strathclyde, 161 Cathedral Street, Glasgow G4 0RE, U.K.; ⊥ LifeScan Scotland Ltd, Beechwood Park North, Inverness IV2 3ED, U.K.; # NHS GGC, Department of Microbiology, Glasgow Royal Infirmary, NEW Lister Building, Glasgow G31 2ER, United Kingdom; ¶ School of Medicine, Dentistry & Nursing, College of Medical Veterinary & Life Sciences, University of Glasgow, Glasgow G12 8QQ, United Kingdom; ∞ Department of Pure and Applied Chemistry, University of Strathclyde, 295 Cathedral Street, Glasgow, G1 1XL, United Kingdom

## Abstract

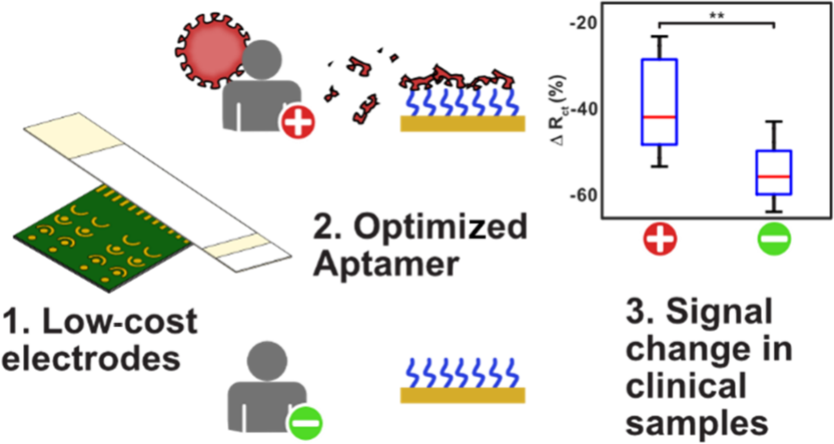

SARS-CoV-2 diagnostic practices broadly involve either quantitative polymerase chain reaction (qPCR)-based nucleic amplification of viral sequences or antigen-based tests such as lateral flow assays (LFAs). Reverse transcriptase-qPCR can detect viral RNA and is the gold standard for sensitivity. However, the technique is time-consuming and requires expensive laboratory infrastructure and trained staff. LFAs are lower in cost and near real time, and because they are antigen-based, they have the potential to provide a more accurate indication of a disease state. However, LFAs are reported to have low real-world sensitivity and in most cases are only qualitative. Here, an antigen-based electrochemical aptamer sensor is presented, which has the potential to address some of these shortfalls. An aptamer, raised to the SARS-CoV-2 spike protein, was immobilized on a low-cost gold-coated polyester substrate adapted from the blood glucose testing industry. Clinically relevant detection levels for SARS-CoV-2 are achieved in a simple, label-free measurement format using sample incubation times as short as 15 min on nasopharyngeal swab samples. This assay can readily be optimized for mass manufacture and is compatible with a low-cost meter.

The SARS-CoV-2 virus, which gives rise to the disease COVID-19, has caused widespread disruption of normal societal activities and a high mortality of nearly 5.3 million people in the last 2 years.^1,2^ An important aspect of the response to the SARS-CoV-2 pandemic has been rapid testing.^3−5^

Reverse transcriptase quantitative polymerase chain reaction (RT-qPCR) has been the mainstay of testing during the pandemic due to its high sensitivity. However, the test requires expensive instrumentation, laboratory infrastructure, and trained personnel to complete the test. Furthermore, the small viral RNA fragments detected using RT-qPCR can lead to residual viral traces being erroneously identified as an ongoing infection.^6−9^ Some studies have suggested that threshold cycle, *C*_T_, could be used to inform the quarantine period and infer the viral load.^10^ However, measuring contagiousness using this method is potentially misleading due to differences in sample variability, C_T_ variation across samples, and variation in the physiological state of patients.^11^

An alternative nucleic acid amplification technique is the use of Loop-mediated isothermal AMPlification (LAMP), which holds promise as a selective and sensitive test with the potential for point-of-need use. Several approaches have been described recently for SARS-CoV-2.^12^ To date, however, few have been commercially adopted. An example of a commercial LAMP test approved for use in the UK is the OptiGene COVID-19_RT-LAMP kit. This kit has a very high sensitivity but requires the Genie fluorescence measuring instrumentation and is only CE marked for professional use in laboratories.^13,14^

Antigen-based lateral flow assays (LFAs) have the potential to deliver faster test results, but they are principally qualitative and were shown to exhibit SARS-CoV-2 sensitivities ranging from 37 to 99% when analyzed in a recent systematic review.^15^ Furthermore, although a preliminary performance evaluation report on LFA testing in the UK highlighted a high degree sensitivity of 79% when used by laboratory staff, this dropped to 58% when used by self-trained members of the public.^16^ LFA accuracy has also been related to viral load, with sensitivity dropping to 40% for viral loads of less than approximately 100 RNA copies/mL.^17^

Electrochemical biosensors hold promise for achieving widespread, low-cost, and multiplexed diagnosis of infectious and complex diseases. Numerous examples exist of successful development and characterization of sensitive and selective electrochemical biosensors.^18−21^ Electrochemical impedance spectroscopy (EIS) is particularly attractive because of its non-destructive nature, high sensitivity, and potential for realization of label-free measurements, leading to simple assay workflows.^22,23^ Faradaic EIS, that is, measurements in the presence of a redox couple, has been shown to be particularly effective for the identification of binding at biofunctionalized electrodes. This method works through measurement of changes in the charge-transfer resistance (*R*_ct_) where electron transfer is impeded by the binding and accumulation of a target analyte at the sensor surface. This technique could be combined with low-cost electrodes such as those produced for the blood glucose industry, which have a commercial unit cost of around £0.20 per test and can be produced in quantities of several million per day.^24,25^

Aptamers are synthetic nucleic acid sequences selected for their affinity to specifically bind molecular targets of choice, including small molecules, proteins, and pathogens. They are selected from a large library of randomly generated sequences through iterations of increasing selective pressure. They have numerous advantages over other affinity binding agents such as antibodies, including chemical stability (improved shelf life), the ability to synthesize large quantities at a relatively low cost, and the easy functionalization with chemical groups for labeling, surface immobilization, or detection.^22,26−28^ Consequently, they are very compatible with electrochemical measurements. The further optimization of selected primary aptamer sequences enables even higher affinities between the aptamer-coated probe and the target.^29^

This work presents an impedimetric SARS-CoV-2 biosensor using SARS-CoV-2 truncated aptamers, compatible with low-cost electrode systems, both of which elements are highly scalable. The results show that following the successful development of an anti-spike protein truncated aptamer, sensitive and selective detection of the recombinant SARS-CoV-2 spike protein can be achieved. Using the developed biosensor assay, it is possible to discriminate between positive and negative SARS-CoV-2 patient samples with a 15 min sample incubation step. The results demonstrate the possibility of a SARS-CoV-2 biosensor that can be produced at scale, with an ultra-low reagent cost (the aptamer reagent is estimated to cost £0.01–0.03 UK pence per test) and which can be read out using established potentiostat circuits from blood glucose monitoring for low-cost, rapid, and highly sensitive diagnostics.

## Experimental Section

### Reagents

Thin-film gold electrodes (TFGEs), used as the electrode substrate in blood glucose test strips, were supplied by LifeScan (Inverness, UK). Aptamers and their buffer system were developed and synthesized by Aptamer Group (York, UK). Molecular biology water, Cytiva illustra NAP Columns (NAP-10), potassium ferricyanide, potassium ferrocyanide, potassium chloride, and sodium sulfate were obtained from Fisher Scientific (Loughborough, UK). Phosphate buffer saline (PBS) and deionized water were purchased from Scientific Laboratory Supplies Limited (Nottingham, UK). Horseradish peroxidase (HRP)-tagged SARS-CoV-2 Spike Glycoprotein S1 (HRP-S1) was obtained from the Native Antigen Company (Kidlington, UK). Abcam (Cambridge, UK) supplied the recombinant human IL-6. Alfa Aesar (Ward Hill, MA, USA) supplied the 4-morpholinoethanesulfonic acid buffer. Merck (Dorset, UK) supplied tris(2-carboxyethyl)phosphine hydrochloride (TCEP), bovine serum albumin (BSA), magnesium chloride, calcium chloride, sodium chloride, and Tween.

### Aptamer Production

Aptamer discovery and development was performed by Aptamer Group (York, UK) according to proprietary selection methods.^30−32^ These aptamers are commercially known as Optimer binders. Briefly, the SARS-CoV-2 S1 domain of the Spike protein (Sino Biological, Eschborn, Germany) was immobilized onto nickel beads via His-tags and used for DNA aptamer selection from a library of 10^14^ sequences via eight successive rounds of *in vitro* selection. Following selection, the aptamers were further optimized for performance by determining the smallest fragment of the parent aptamer that yielded the required target binding profile. Twenty-three different fragment molecules were generated from the parent aptamer, consisting of reductions in size between 20 and 80%. The truncated aptamers were assessed and validated by bio-layer interferometry (BLI) using an Octet Red 384 system (Sartorius, Goettingen, Germany): for target affinity, functional binding to the SARS-CoV-2 S1 domain and SARS-CoV-2 spike protein trimer (Peak Protein, Macclesfield, UK) and lack of cross-reactivity to the homologous SARS-CoV and Human Coronavirus NL63 (HCoV-NL63) Spike protein S1 domains (Sino Biological). The resulting parent and truncated aptamer sequences are 81 and 33 nucleotides long, respectively, and their corresponding selection buffer composition is detailed in Table S2. The selected SARS-CoV-2 truncated aptamer was synthesized with a biotin modification at the 5′ terminus to allow for conjugation to the streptavidin-coated biosensors (Sartorius) to assess their binding properties.

For gold electrode immobilization and orientation, both parent and truncated aptamer sequences were modified with a thiol group at their 5′ terminus. Parent aptamers used here required a temperature treatment to reach a functional state. They were denatured at 95 °C for 5 min and then placed at 4 °C for 10 min. The truncated sequences were ready to use as supplied.

### Electrode Fabrication

TFGEs were fabricated by LifeScan (Inverness, UK) by sputter-coating gold onto a polyester substrate. A polymer-adhesive layer containing a 1.17 mm pre-cut channel was attached to the gold-coated polyester. The substrate was then cut into 3.5 mm wide electrodes, resulting in a bilaminate structure with 1.17 × 3.5 mm gold working electrodes. The overall width of the polyester/adhesive roll was less than the gold film roll, thus ensuring that a second area of the exposed gold film was present for electrical contact purposes (Figure 2A). Batches of 50 electrodes were packaged in vials containing desiccant material until required for use. Prior to functionalization, the electrodes were rinsed with deionized water and air-dried.

### Electrode Functionalization

All reactions were carried out at ambient temperature. BSA was added to folded parent aptamers and truncated sequences to reach a final concentration of 0.01% in solution. Aptamers were then reduced for 45 min with 1 mM TCEP to remove dithiol bonds at the 5′ end and later filtered using NAP-10 columns to 30 and 32.4 nM for parent and truncated sequences, respectively. The NAP-10 columns were used to remove residual TCEP and minimize any cross-reactivity. The aptamers were then immobilized onto bare electrodes for an hour. When stated, the remaining free surfaces were blocked with 0.1% BSA for further 60 min. After surface functionalization, the electrodes were rinsed with PBS, and EIS measurements were taken.

### Assay Protocols

Following electrode functionalization, 80 ng mL^–1^ of HRP-S1 in the selection buffer was incubated on the selected electrodes for 30 min or 80 ng mL^–1^ of recombinant IL-6 in the selection buffer for negative control conditions. Following a PBS rinse, EIS measurements were taken.

### Clinical Sample Testing

The electrode functionalization and format were adapted to university and hospital access and restrictions, equipment knowledge, and personnel availability limitations due to the pandemic. SEP1 BIOTIP multichannel electrode printed circuit boards (PCBs) (Bath, UK) were used for clinical sample testing and prepared according to the cleaning procedure described by Vezza et al. (2021).^33^ Truncated aptamers were reduced, filtered, and co-immobilized with BSA onto PCB working electrodes for 60 min as described in the electrode functionalization paragraph above. The selection buffer was used for rinsing and complete immersion of functionalized electrodes overnight and during transportation. Positive and negative SARS-CoV-2 samples were obtained from combined oropharyngeal and nasal swabs at NHS Glasgow Royal Infirmary and immediately deactivated in viral PCR sample solution (VPSS) upon sampling. EIS was measured the following day, before and after a 15 min exposure to clinical samples in 5 mM [Fe(CN)_6_]^3–/4–^ in a background of the selection buffer.

### Electrochemical Measurements and Data Processing

A platinum counter (Metrohm, Runcorn, UK) and 3 M NaCl Ag/AgCl reference (IJ Cambria, Llanelli, UK) electrodes were used to perform all measurements with TFGEs in 5 mM [Fe(CN)_6_]^3–/4–^ in PBS. Cyclic voltammetry (CV) measurements were obtained by sweeping a potential range from −0.4 V to +0.6 V at 0.1 V s^–1^. Differential pulse voltammetry (DPV) measurements mirrored the CV potential window and scan rate, with a respective potential pulse and time of 0.025 V and 0.05 s. EIS was measured after 10 min of stabilization in [Fe(CN)_6_]^3–/4–^ against an open-circuit potential from 100 kHz to 0.1 Hz. *E*_ac_ set at 0.01 *V*_rms_ and *E*_dc_ at 0 V. 67 frequencies were recorded for characterization and 50 frequencies for all other experiments.

A PalmSens4 potentiostat and PSTrace software from Palmsens BV (Houten, the Netherlands) were used to perform all electrochemical measurements. Nyquist plots were then fitted to a modified Randles’ equivalent circuit. Subsequent parameters of interest were extracted and analyzed using Origin and Matlab.

## Results and Discussion

### Bio-Layer Interferometry Analysis of the SARS-CoV-2 Truncated Aptamer

**Figure 1:**
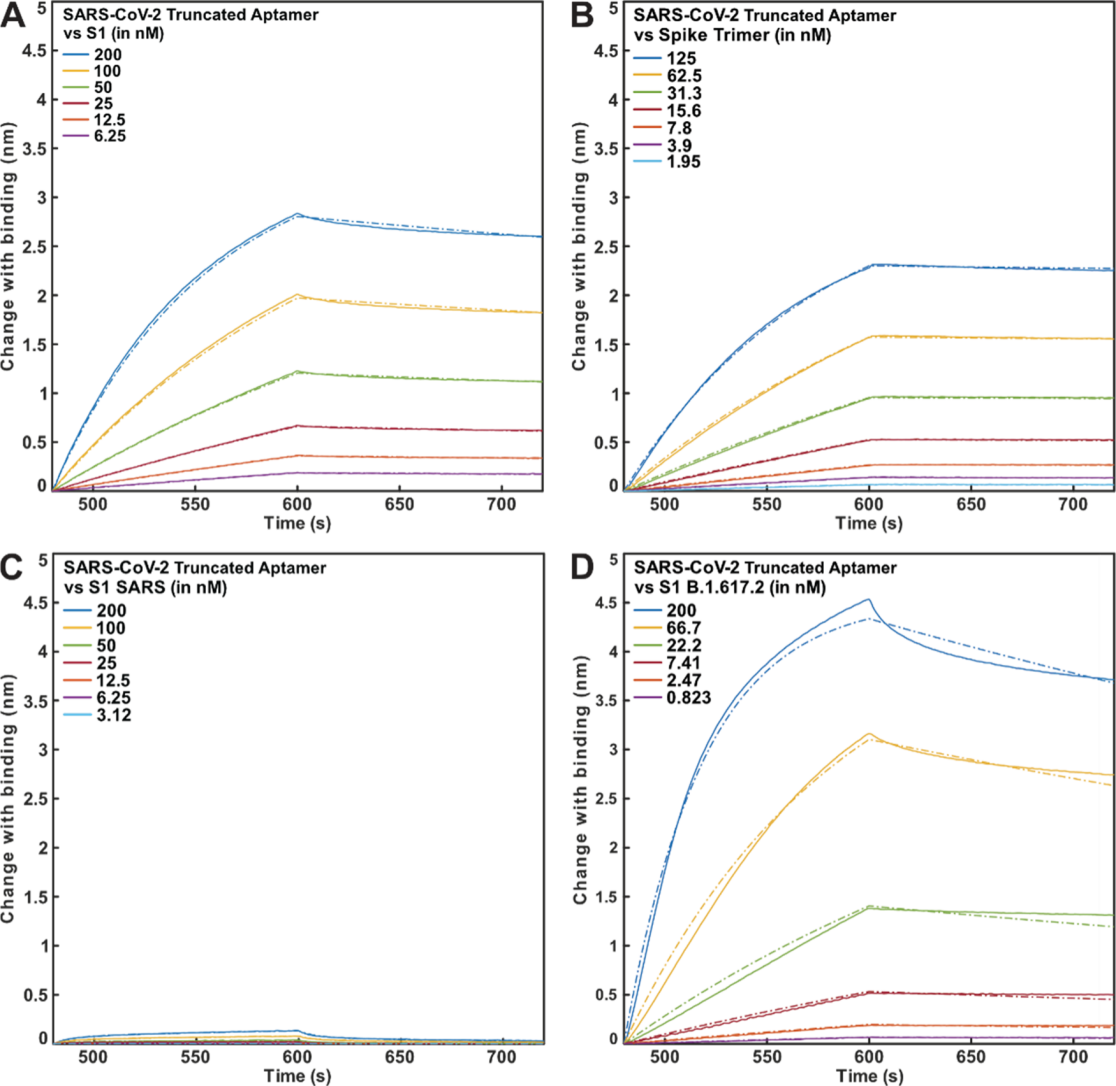
BLI data (full lines) of the SARS-CoV-2 aptamer against various concentrations of SARS-CoV-2 WT S1 (A), SARS-CoV-2 WT spike trimer (B), SARS-CoV S1 (C), and S1 of the B.1.617.2 variant (D), all fitted to a 1:1 binding model.

The SARS-CoV-2 S1 truncated aptamer was immobilized onto streptavidin-coated biosensors via a biotin group at the 5′ terminus. Affinity was subsequently determined by monitoring the interaction between the truncated aptamer and the (i) SARS-CoV-2 S1 protein domain and the (ii) SARS-CoV-2 spike protein trimer. The selected truncated aptamer was shown to bind both the singular S1 domain and the trimeric receptor (Figure 1A,B) with low nanomolar affinity (Table 1). No interaction was observed between the control scrambled truncated aptamer and spike protein trimer, confirming the specificity of the binder (Figure S4). The specificity of the aptamer was determined via analysis of binding to homologous coronavirus S1 recombinant protein domains of the SARS-CoV (Figure 1C) and HCoV-NL63 (Figure S1). The observed minimal shift in wavelength for the truncated aptamer in the presence of the homologous coronavirus proteins indicates minimal to no target binding and high specificity to the SARS-CoV-2 target. Due to the continuous evolution of the SARS-CoV-2 virus, further interaction analysis was performed to assess binding to the S1 domain of three SARS-CoV-2 variants of concern (B.1.1.7, B.1.351, and B.1.617.2). The aptamer sequence showed effective binding to each of the SARS-CoV-2 variants (Figures 1D, S2, and S3; Tables 1 and S1), demonstrating that the S1 protein binding site of the truncated aptamer is independent of the site of S1 mutations.

**Table 1:** Affinities between the SARS-CoV-2 Truncated Aptamer and the Tested Targets

protein	KD (nM)
SARS-CoV-2 WT S1 domain	10.17 ± 0.07
SARS-CoV-2 WT trimer	1.19 ± 0.04
SARS-CoV S1 domain	no binding determined
SARS-CoV-2 B.1.617.2 S1 domain	11.07 ± 0.1

### Electrochemical Characterization of TFGEs

**Figure 2:**
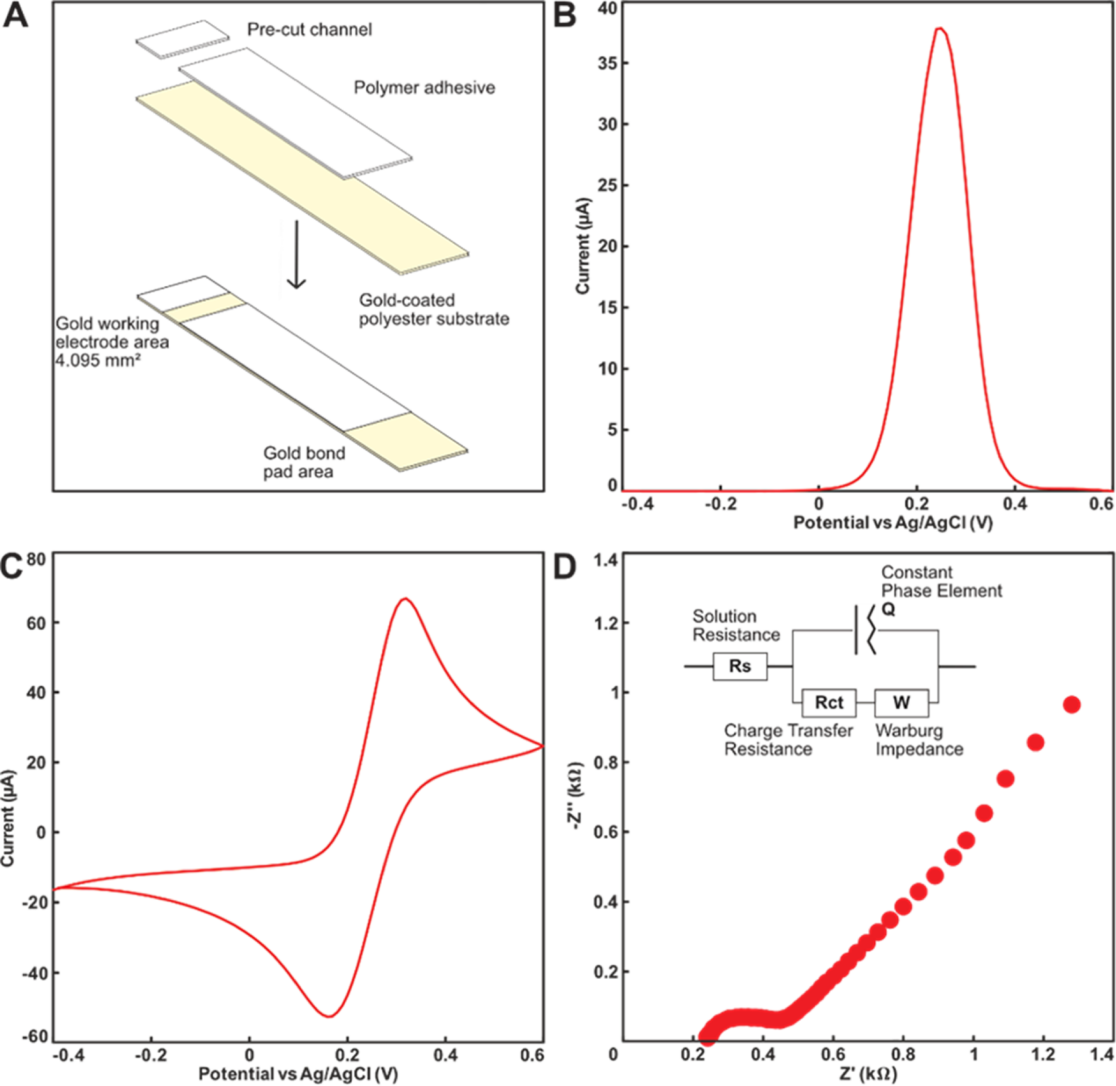
Characterization of TFGEs, (A) fabrication and (B) DPV, (C) CV, and (D) EIS responses in 1 × PBS containing 5 mM [Fe(CN)_6_]^3–/4–^ and modified Randles equivalent circuit as the inset.

Prior to functionalizing with aptamers and testing the responsiveness of the electrodes, it was necessary to perform a basic characterization to understand and quantify the electrochemical performance of the TFGE sensors. The TFGE sensors came packaged with an upper release liner above the electrode area to prevent accumulation of dust particles and adsorption of organic residues to the gold surface that was removed before characterization (Figure 2A). Due to the very thin gold film (5–10 nm) and the cleanliness of the electrodes as shipped, it was found that surface pre-treatment was not required and the electrodes could be either characterized or functionalized without any prior treatment. Characterization carried out in 1 × PBS containing 5 mM [Fe(CN)6]^3–/4–^ indicates a clean electrode surface with Faradaic charge transfer in DPV (Figure 2B), CV (Figure 2C), and EIS (Figure 2D) measurements. To evaluate the performance of the sensors and check the integrity of the dielectric on the sensor, the results from CV and EIS were interpreted using the following two equations, respectively
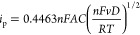
1
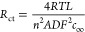
2where *i*_p_ is the voltammetric peak current, *F* is Faraday’s constant, *R* is the universal gas constant, ν is the scan rate, *n* is the number of electrons transferred, *R*_ct_ is the charge transfer resistance, *A* is the electrode area, *D* is the diffusion coefficient, and *c*_∞_ is the bulk concentration of the redox agent.

The peak height from CV experiments was used to calculate a diffusion coefficient of 1 × 10^–5^ cm^2^ s^–1^ from eq 1 for potassium ferricyanide. This is consistent with other reported examples and confirms the integrity of the passivation layer and performance of the electrodes. Additionally, the fitted value of *R*_ct_ was found to be 208 Ω. This is close to the theoretical value of 186 Ω from eq 2 for an ideal electrode. In combination, these two calculations provided confidence that the sensors as fabricated were well defined by the passivation material and that electron transfer and transport processes were suitable for use without further cleaning procedures prior to biological functionalization. This is an attractive advantage as a cleaning step is challenging to implement in an assay production line.

### Electrochemical Measurement of SARS-CoV-2 Using Aptamer-Modified Electrodes

**Figure 3:**
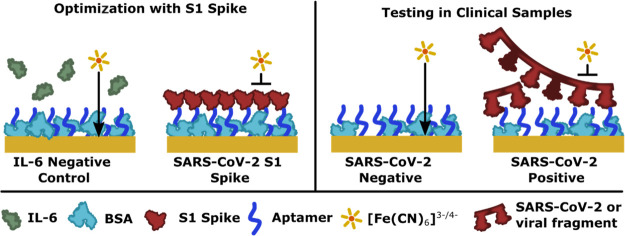
Depiction of the surface functionalization and the nature of the impedimetric response following immobilization of the aptamer and upon binding of either the recombinant spike protein or the SARS-CoV-2 patient samples.

The performances of both the parent aptamer (81 nucleotides) and the truncated aptamer (33 nucleotides) were assessed on the TFGE strips in a biosensor format, in combination with a BSA surface backfilling step (Figure 3). BSA was chosen as an inexpensive, non-specific adsorption agent, and as it was part of the aptamer selection process, it was ensured that it would not cross-react. BSA’s passive physical adsorption onto the gold surfaces integrates well with the sensor development.^34^ In addition to testing both aptamers on a BSA backfilled surface, the truncated sequence was tested following immobilization onto the gold surface and without any backfilling step. This gave rise to three biosensor configurations: parent aptamer plus BSA; truncated aptamer plus BSA; and truncated aptamer without BSA.

**Figure 4:**
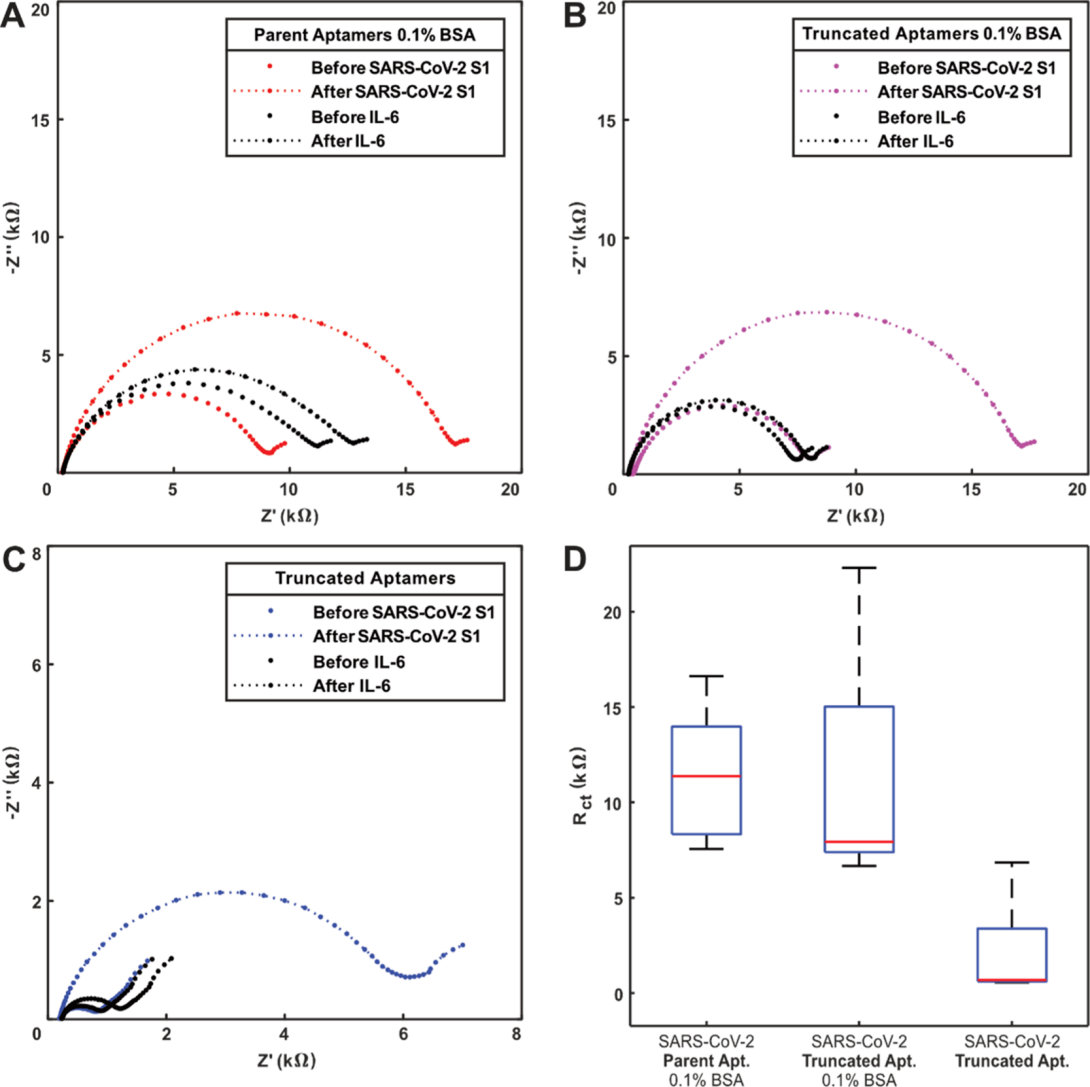
Nyquist plots of each biosensor configuration before and after exposure to 80 ng mL^–1^ of SARS-CoV-2 S1 or IL-6 (*n* = 1 for each trace). (A) Parent aptamer-modified electrodes with BSA, (B) truncated aptamer-modified electrodes with BSA, (C) truncated aptamer-modified electrodes without BSA, (D) summary box plots showing starting *R*_ct_ values for all three surface modifications (*n* = 6, median in red and minimum to maximum value range for the whiskers).

Next, the response of the electrode to the SARS-CoV-2 S1 protein was explored. In all configurations tested, *R*_ct_ increased more than twofold when SARS-CoV-2 S1 was presented to the electrode surface compared to a much smaller change on incubation with IL-6. The results demonstrate that the interaction between both parent and truncated aptamers and the spike protein was much stronger following binding with SARS-CoV-2 S1 than following incubation with the IL-6 protein (Figure 4A–C).

Adding the BSA antifouling layer has the effect of increasing the baseline *R*_ct_ (Figure 4D). This is expected and is related to the relative molecular weight difference between aptamer sequences and BSA.

Interestingly, the parent aptamer with BSA showed the largest initial *R*_ct_ value, the truncated aptamer with BSA showed an intermediate value, and the truncated aptamer without any BSA showed the lowest *R*_ct_. These results show that the layers immobilized onto the electrode are behaving reliably and predictably and that surface functionalization was working as intended.

**Figure 5:**
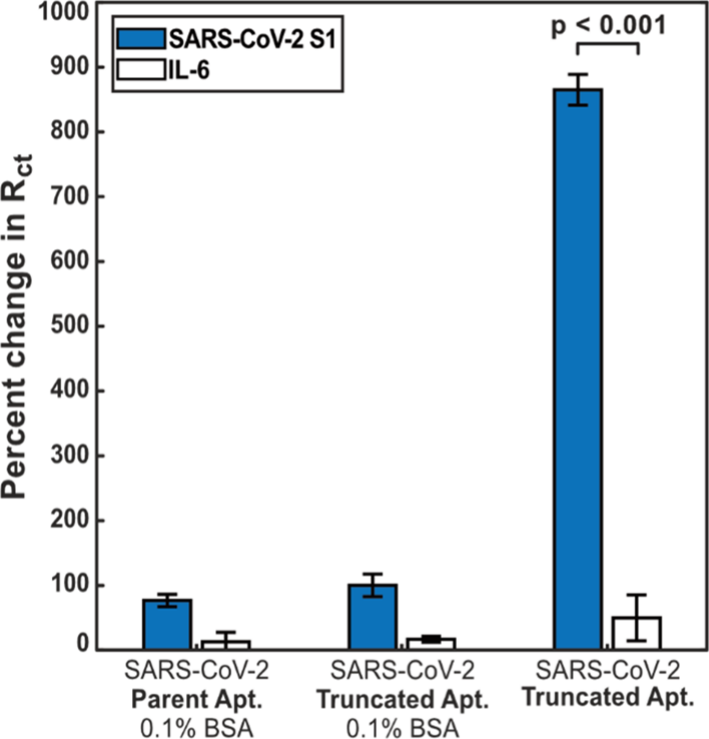
Assay signal increase responses following exposure to 80 ng mL^–1^ SARS-CoV-2 S1 and IL-6 proteins for electrode surfaces modified as follows: parent aptamer + 0.1% BSA, truncated aptamer + 0.1% BSA, and truncated aptamer only.

All three-electrode configurations were then tested with solutions of the SARS-CoV-2 S1 spike protein and IL-6 as negative control. IL-6 was chosen as a negative condition because the aptamer specificity was already demonstrated during selection against spike proteins from other coronaviruses and flu antigens and because IL-6 production is triggered in many inflammatory diseases^35^ that could result in non-specific adsorption onto electrodes when present in the sample. The sensor based upon the truncated aptamer sequence, incubated without BSA, gave the highest signal change of the three modifications tested, with a contrasting change in signal from the S1 spike protein and IL-6. For all three sensor configurations tested, the S1 spike protein binding was much greater than for the IL-6 negative control, showing that it was possible to both specifically detect the viral protein of interest at a concentration of 80 ng mL^–1^ (Figure 5).

It is interesting to discuss the pronounced signal change between S1 and IL-6 when BSA is not included as an anti-fouling layer. This finding is likely to be due to the nature of the initial surface modification. In the truncated aptamer-only configuration, the lower concentration of BSA on the electrode surface provides a higher baseline current flux in contrast to configurations with additional BSA for anti-fouling. This is consistent with similar detection schemes, such as the use of nucleic acid probe structures with both morpholino and PNA probes showing higher overall analytical sensitivity due to lower starting impedances that provide greater resolution of target binding.^36,37^

### Testing in Clinical Samples

Limited access was available to clinical samples. Due to biosecurity and sample size constraints, LifeScan TFGEs were supplanted for a gold multiplexed format (Biotip PCBs), which we had previously used to produce a low-cost SARS-CoV-2 sensor based on ACE2^33^ and had been validated for use in the heightened biosecurity environment at the hospital. This sensor type allowed a higher throughput of testing per sample by utilizing multiple electrodes within each well. The truncated aptamer without BSA was used for these measurements, as this provided the greatest impedance change upon binding of SARS-CoV-2 S1. The PCBs were functionalized with TCEP treated and NAP-10-filtered truncated aptamers and then immersed in the selection buffer until testing. EIS was measured in the selection buffer containing 5 mM [Fe(CN)_6_]^3–/4–^ before and after exposure of each PCB to either a SARS-CoV-2-positive (*C*_T_ = 26) or SARS-CoV-2-negative patient sample.

**Figure 6:**
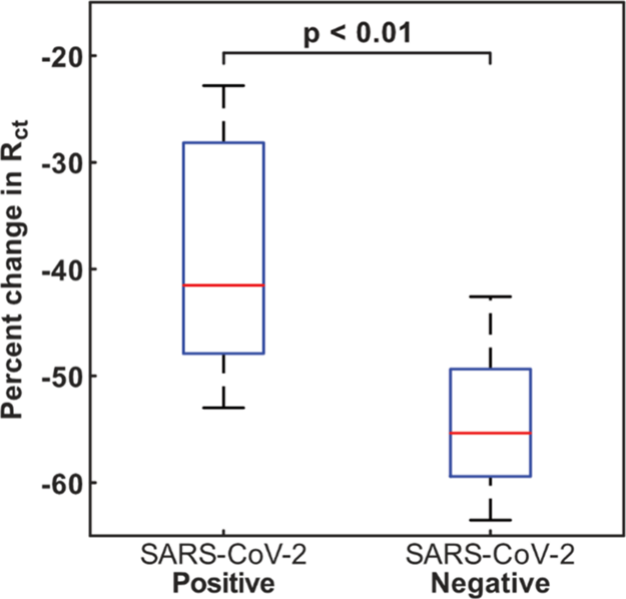
Assay signal change after 15 min of exposure to either a SARS-CoV-2-positive (*C*_T_ = 26) or a SARS-CoV-2-negative patient sample deactivated in VPSS (*n* = 8 for each sample).

Combined oropharyngeal and nasal swab clinical samples were provided for testing by a local hospital. Despite nasopharyngeal swabs being the gold standard for the diagnosis of SARS-CoV-2 infection because of higher sensitivity, combined oropharyngeal and nasal samples offer an alternative with similar sensitivity when personal protective equipment and testing supplies are limited and repeated screening is necessary.^38,39^ PCBs were rinsed after sample incubation with the selection buffer. This was carried out because the selection buffer contains Tween, thus enabling the potential for non-specific binding from the complex sample matrices (Figure 6).

It should also be noted that the results with clinical samples show a further modification to the electrochemical signal, from an increase to a decrease in the *R*_ct_ signal (Figure 6). This was attributable to the VPSS inactivation medium, which contains a surfactant which affects the wetting properties of the electrode surface and reduces the *R*_ct_. This explains the general reduction in impedance, above which the true assay signal extends. Figure S5 shows this phenomenon, with a clear demonstration of how 1% Triton X when incubated on the electrode surface (clean and SAM-modified) causes a generalized decrease in the charge transfer resistance. In spite of the effect of the clinical sample medium on the sensor surface, it was possible to clearly distinguish between positive and negative samples (Figure 6). Furthermore, the drop in impedance is entirely consistent with findings previously reported by the group using ACE2-modified gold surfaces^33^ and the same clinical sample handling media.

## Conclusions

This work shows the development of a specific aptamer sequence for the SARS-CoV-2 spike protein and its subsequent use for the detection of the virus from complex clinical samples. The detection system as presented has several key advantages including a simple impedance measurement to determine target binding, a high stability aptamer receptor to detect the spike protein, and the use of low-cost gold electrodes, similar to blood glucose sensors. Furthermore, the technology has the potential to scale up and would unlock the production volumes inherent in, for example, the diabetes test strip manufacturing industry. Production and functionalization at high volumes of all components of the aptasensor would meet the ongoing demand for SARS-CoV-2 mass testing.

## Supporting Information Available

Additional experimental details on the aptamer BioLayer Interferometry data and processing (PDF)
