# Supplementary material for: SARS-CoV-2 Aptasensors Based on Electrochemical Impedance Spectroscopy and Low-Cost Gold Electrode Substrates
Source: Anal Chem. 2022 Jan 19;94(4):2126–33. doi: 10.1021/acs.analchem.1c04456 (PMC8790822; doi:10.1021/acs.analchem.1c04456)
Supplement: Supplementary file 1 — ac1c04456_si_001.pdf [file ac1c04456_si_001.pdf]

Supporting Information

A SARS-CoV-2 aptasensor based on electrochemical impedance spectroscopy and low-cost gold electrode substrates.

Perrine Lasserre[a], Banushan Balansethupathy[b], Vincent J. Vezza[a], Adrian Butterworth[a], Alexander Macdonald[a], Ewen O. Blair[a], Liam McAteer[a], Stuart Hannah[a], Andrew C. Ward[c], Paul A. Hoskisson[d], Alistair Longmuir[e], Steven Setford[e], Eoghan C. W. Farmer[f], Michael E. Murphy[f,g], Harriet Flynn[b] and Damion K. Corrigan[a]*.

[a] Department of Biomedical Engineering, University of Strathclyde, 106 Rottenrow East, Glasgow G4 0NW, United Kingdom.

[b] Aptamer Group, Suite 2.78–2.91, Bio Centre, Innovation Way, Heslington, York YO10 5NY, United Kingdom.

[c] Department of Civil and Environmental Engineering, University of Strathclyde, 75 Montrose Street, Glasgow G1 1XJ, United Kingdom.

[d] Strathclyde Institute of Pharmacy and Biomedical Sciences (SIPBS), University of Strathclyde, 161 Cathedral St, Glasgow G4 0RE, United Kingdom.

[e] LifeScan Scotland Ltd, Beechwood Park North, Inverness IV2 3ED, United Kingdom.

[f] NHS GGC, Glasgow Royal Infirmary, Department of Microbiology, NEW Lister Building, Glasgow G31 2ER, United Kingdom.

[g] School of Medicine, Dentistry & Nursing, College of Medical Veterinary & Life Sciences, University of Glasgow, Glasgow G12 8QQ, United Kingdom.

CORRESPONDENCE

Damion K. Corrigan - damion.corrigan@strath.ac.uk

CONTENTS

Processing and analysis of SARS-CoV-2 aptamer binding data by Bio-layer Interferometry
Supporting Figures S1-S4
Supporting Table S1

Selection buffer composition
Supporting Table S2

Triton-X verification on gold
Figure S5

PROCESSING AND ANALYSIS OF APTAMER BINDING DATA BY BIO-LAYER INTERFEROMETRY

Binding sensorgrams of Aptamer reagents to the recombinant proteins were first aligned at the start of the analyte binding phase and reference subtracted with a probe against buffer alone. The data was then fitted to a 1:1 Langmuir binding model using the Octet Data analysis HT 12.0.2.59 software. In this model, both the association and dissociation phases exhibit a time-resolved signal that is defined by a single exponential function. It is assumed that the analyte molecules bind at the same rate to every binding site on the ligand. The association curve shows an exponential increase in signal followed by a levelling off to plateau as the binding reaching equilibrium and the dissociation curve follows a single exponential decay with the signal returning to baseline as the complex dissociates. A global fit was performed on the SARS-CoV-2 aptamer affinity data as this gives the most accurate kinetic and affinity constants using several analyte concentrations. The global fit analysis utilises all binding curve data in the group using a full fit option. Fitting of multiple concentration curves to one set of results yields more robust and reliable curve fits.

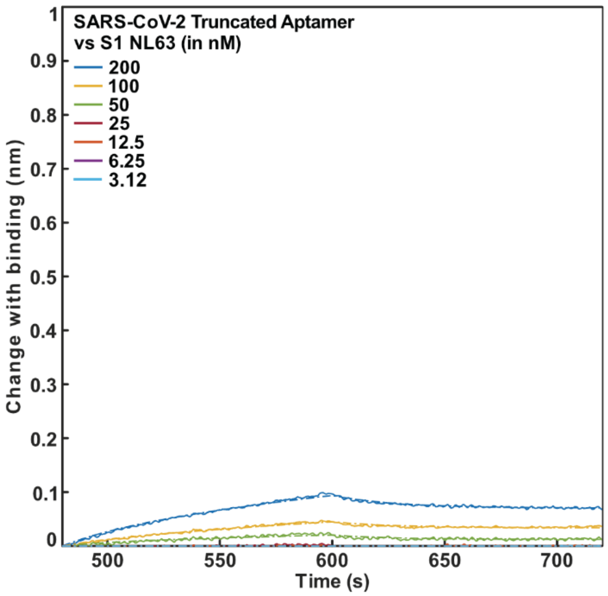

Figure S1. Bio-Layer Interferometry data (full lines) of the SARS-CoV-2 aptamer against different concentrations of a common flu virus NL63 fitted to a 1:1 binding model (dotted lines).

Table S1. Affinities between the SARS-CoV-2 aptamer and tested targets.

Protein	KD (nM)
**HCoV-NL63 S1 Domain**	No binding determined
**SARS CoV-2 B.1.1.7 S1 Domain**	13.50 ± 0.25
**SARS CoV-2 B.1.351 S1 Domain**	6.71 ± 0.22

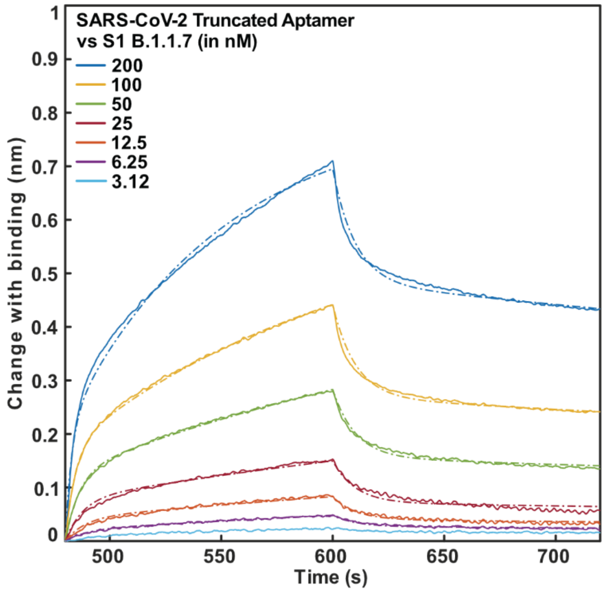

Figure S2. Bio-Layer Interferometry data (full lines) of the SARS-CoV-2 aptamer against different concentrations of the SARS-CoV-2 variant B.1.1.7 fitted to 1:1 binding model (dotted lines).

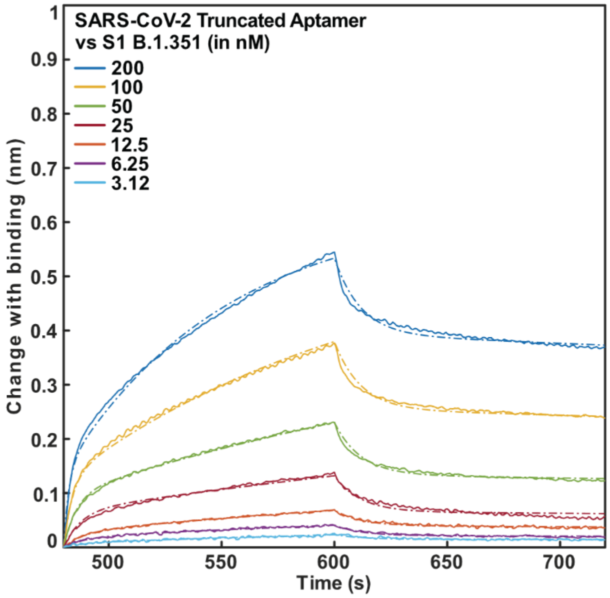

Figure S3. Bio-Layer Interferometry data (full lines) of the SARS-CoV-2 aptamer against different concentrations of the SARS-CoV-2 variant B.1.351 fitted to 1:1 binding model (dotted lines).

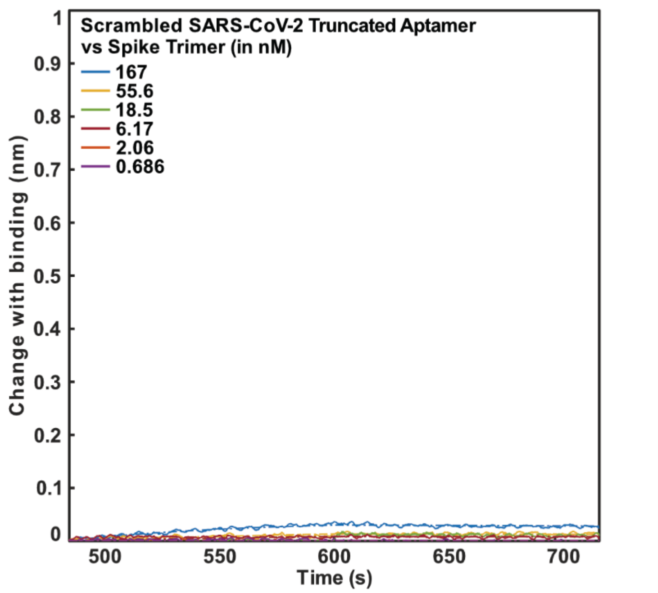

Figure S4. Bio-Layer Interferometry data (full lines) of a scrambled version of the SARS-CoV-2 truncated aptamer against different concentrations of the SARS-CoV-2 trimer fitted to 1:1 binding model (dotted lines).

Table S2. Selection buffer composition.

Concentration	Reagent
**50 mM**	MES buffer pH 6.2
**5 mM**	$MgCl_2$
**1 mM**	$CaCl_2$
**220 mM**	NaCl
**4.50 mM**	KCl
**20 mM**	Na_2SO_4
**0.01%**	Tween
Optional:	
**0.01%**	BSA

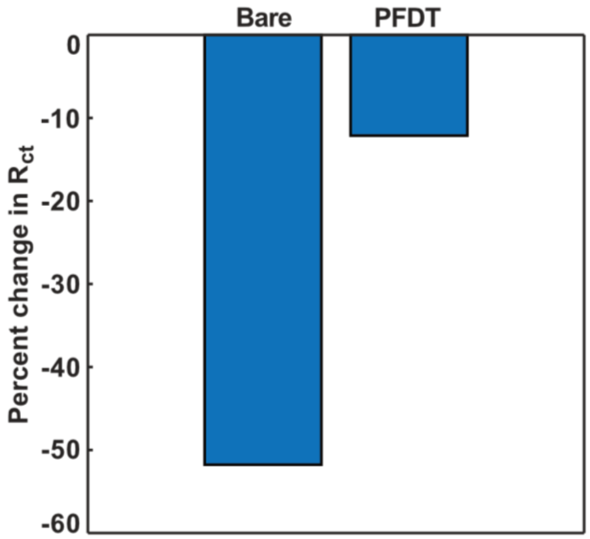

Figure S5. Signal change in charge transfer resistance after a 15-minute incubation in 1% Triton-X of bare gold electrodes (*n*=2) and functionalised gold electrodes with 1 mM of 1H,2H,2H,2H-Perfluorodecanethiol (*n=3).*
